# Editorial: Percutaneous Mitral Valve Interventions (Repair): Current Indications and Future Perspectives

**DOI:** 10.3389/fcvm.2020.581109

**Published:** 2020-11-03

**Authors:** Alfonso Ielasi, Andrea Buono, Fabien Praz, Azeem Latib

**Affiliations:** ^1^Clinical and Interventional Cardiology Unit, Istituto Clinico S. Ambrogio Gruppo Ospedaliero San Donato, Milan, Italy; ^2^Cardiology Department, Inselspital, Bern University Hospital, University of Bern, Bern, Switzerland; ^3^Department of Cardiology, Montefiore Medical Center, New York, NY, United States

**Keywords:** mitral regurgitation, trans-catheter mitral valve repair, heart failure, structural heart intervention, mitral

Mitral regurgitation (MR) is one of the most common valvular heart diseases in both European and U.S. populations, with its prevalence increasing with age (1). Severe MR strongly and negatively impacts prognosis, causing chronic left ventricle (LV) volume overload that culminates over time in the irreversible dilation and dysfunction of cardiac chambers. For this reason, delivering a timely treatment for symptomatic patients with moderate-to-severe MR represents a therapeutic priority.

To date, no single treatment option for MR could be considered the gold-standard, since disease- and patient-related characteristics can differ widely. Historically, surgery [mitral valve (MV) replacement or repair] has been the therapeutic cornerstone for MR, and is still considered the first-line therapy for patients with MR. However, in recent years, progressive technological improvements in the field of interventional cardiology have allowed us to approach MR with different trans-catheter techniques (mainly targeting the MV leaflets), which offer the benefits of being less invasive and having shorter patient recovery times. These advantages translate in a therapeutic alternative to conventional surgery for high-risk surgical or inoperable MR patients.

The knowledge that the MV is a complex anatomical apparatus has shed light on the etiology of functional MR (FMR), a disease primarily of the LV and/or atrium. Due to the lack of strong evidence concerning surgical benefit (2, 3), treatment of FMR still represents an unmet clinical need. In this setting, percutaneous interventions are considered a valid therapy in symptomatic patients. Different types of transcatheter treatments have been developed. Most of the available evidence is derived from MitraClip (Abbott Vascular, Abbott Park, Illinois, USA), the most used and studied percutaneous edge-to-edge repair system. In fact, the *Endovascular Valve Edge-to-Edge REpair Study* (EVEREST II) trial demonstrated MitraClip safety and efficacy in a cohort of MR patients [~75% degenerative (DMR) and ~25% FMR] when compared to conventional surgery (4). Hence, MitraClip is often considered the first transcatheter therapeutic option in both DMR and FMR for patients deemed unsuitable for cardiac surgery. However, recent randomized controlled trials (RCTs) focusing only on FMR provided conflicting results concerning MitraClip efficacy over medical therapy at 2 years: the *COAPT* trial (5) showed clear mortality and heart failure hospitalization rate reductions in patients treated with MitraClip, whereas the *MITRA-FR* trial (6) did not. Many explanations have been postulated for these diverging results, with the most reliable represented by the different stages of FMR patients studied: *MITRA-FR* enrolled patients with more remodeled and dysfunctional LV as well as less severe MR as compared to the *COAPT* population. This aspect underlies the pivotal need to treat FMR before patients enter an advanced “too-late” disease stage. New insight will be derived from the *RESHAPE-HF2* RCT (NCT02444338): 420 patients suffering from symptomatic chronic heart failure with moderate-to-severe or severe FMR and reduced LV ejection fraction will be randomized to either optimal standard of care therapy or MitraClip device plus optimal standard of care therapy. The primary endpoint will consist of a composite rate of recurrent heart failure hospitalizations and cardiovascular death at 24 months. MitraClip is also not the only transcatheter option available. During the last few years, several transcatheter devices have been developed, mimicking different surgical techniques, and many others have started pre-clinical assessment. Beyond MitraClip, indirect and direct MV annuloplasty and chordal replacement systems have been studied. However, to date all these other treatments should still be considered and reported as experimental therapies, because the data was derived from studies with smaller sample sizes and shorter follow-ups.

On the other hand, since the underlying MR mechanism represents a major therapeutic success determinant, a wider therapeutic portfolio will increase the rate of procedural success and durability, reflecting the possibility to select a tailored therapeutic strategy. Transcatheter edge-to-edge repair, indirect and direct MV annuloplasty, and chordal replacement can also be seen as complementary therapeutic options, able to maximize the procedural success. However, large data concerning a combined use are still missing. Undoubtedly, the future of MR treatment will also include transcatheter MV replacement (TMVR). Despite this prediction, transcatheter repair therapies will remain an important part of the therapeutic armamentarium for MR, given their ability to preserve the complex inner MV anatomy. However, transcatheter repair therapies such as MitraClip may close the door to further interventions: once implanted, TMVR will be not feasible anymore. For this reason, the ongoing challenge is to choose the *right* device for the *mechanism* of MR, affecting the *given* patient. To achieve an optimal therapeutic goal, a multidisciplinary assessment of every patient is essential. The referring cardiologist, anesthesiologist, cardiovascular imaging specialist, cardiac surgeon, and interventional cardiologist should all confer on the decision together.

This journal is entirely dedicated to the current indications and future perspectives of percutaneous MV interventions. A comprehensive understanding of MV anatomy, physiology, and pathophysiology (Topilsky) is critical to achieve a successful MR reduction. For this purpose, patient and device selection utilizing a multi-modality cardiac imaging assessment is essential, since well-established feasibility criteria have been provided for several transcatheter devices. Imaging role will be also pivotal in the near future, considering advancements in TMVR (specifically to address its pre-procedural feasibly and prevent left ventricle outflow obstruction) (7). Moreover, although transesophageal echocardiography is now the intra-procedural guide in all interventions (Khalique and Hahn), in the near future intracardiac echocardiography (ICE) will be a concrete potential alternative to transesophageal echocardiography, mitigating the need for endotracheal intubation (8). Detailed overviews of current and future transcatheter systems are provided, ranging from edge-to-edge clips repair (Khan et al.; Shah and Jorde), direct (Gasior et al.), and indirect (Patterson et al.) MV annuloplasty, and chordal repair (Fiocco et al.). Lastly, emerging devices have been analyzed, reporting on available clinical as well as pre-clinical experience (Mangieri et al.) and potential procedural complications (Gheorghe et al.). In conclusion, transcatheter MV repair devices are validated therapeutic options able to accommodate a larger variety of MV anatomies, despite the fact that long-term durability results are still required.

## Author Contributions

All authors listed have made a substantial, direct and intellectual contribution to the work, and approved it for publication.

## Conflict of Interest

The authors declare that the research was conducted in the absence of any commercial or financial relationships that could be construed as a potential conflict of interest.
